# The role of dieting, psychopathological characteristics and maladaptive personality traits in Orthorexia Nervosa

**DOI:** 10.1186/s12888-022-03896-1

**Published:** 2022-04-22

**Authors:** C. Novara, S. Mattioli, S. Piasentin, S. Pardini, E. Maggio

**Affiliations:** Department of General Psychology, University of Padova, Via Venezia 8, 35131 Padova, Italy

**Keywords:** Orthorexia Nervosa, Dieting, Psychopathology, Maladaptive personality traits

## Abstract

**Background:**

Pursuing a healthy diet is not a dysfunctional behavior, but dieting could be an important etiological factor for Orthorexia Nervosa (ON). The aim of this study was to investigate the role of diet in groups with high/low orthorexic tendencies. Moreover, some psychopathological characteristics associated with ON and maladaptive personality traits were investigated.

**Methods:**

The sample consisted of three groups: two were on a diet and had high (HIGH-D; *n* = 52) or low (LOW-D; *n* = 41) orthorexic tendencies. The other was composed of people with high orthorexic tendencies not on a diet (HIGH; *n* = 40). Participants filled out self-report questionnaires to investigate orthorexic tendencies, eating disorders features, obsessive–compulsive symptoms, perfectionism, depressive/anxious symptomatology, and maladaptive personality traits.

**Results:**

The HIGH-D group showed more orthorexic tendencies than the HIGH group. More maladaptive personality traits and anxiety symptoms have been highlighted in HIGH and HIGH-D groups. The HIGH group had more eating disorder characteristics than other groups. Only the HIGH-D group showed more depressive symptoms than the LOW-D group.

**Conclusions:**

The features of HIGH and LOW-D groups suggest that diet alone could not explain ON, even if it could be a possible factor related to ON. Therefore, people with high orthorexic tendencies, psychopathological features, and maladaptive personality traits could be in a prodromic condition for disordered eating habits and deserve clinical attention.

## Background

Orthorexia Nervosa (ON) is a disordered eating characterized by an exaggerated focus on the quality of food in order to control dietary habits in a healthy and proper way. It often begins with the attempt to improve well-being or to avoid/manage health problems. Individuals with high orthorexic tendencies highlight overconcerns about food that could interfere with everyday functioning; most problems regard pervasive preoccupation with food, social isolation, a great amount of time spent to prepare meals or to select/check food, and nutritional deficiencies due to their eating habits [1–3]. Moreover, people with orthorexic symptomatology have high levels of distress when their meals are not in line with their beliefs related to healthy eating; on the other hand, those concerns could lead to other problems [4]. Knowledge about healthy food leads them to criticize other eating habits; moreover, they feel positive emotions due to their eating habits [1]. Although ON is not formally present as a nosological category of the Diagnostic and Statistical Manual of Mental Disorders (DSM-5; [5]) or in other major classification of disorders, many publications have contributed to hypothesize diagnostic criteria [2, 3, 6, 7] in order to consider ON a new psychopathological disorder. Literature highlights important overlaps between ON and the symptomatology of Eating Disorders (ED) [8–11]. There are moderate relationships between ON and obsessive–compulsive features [12, 13]. Non-adaptive perfectionism [13–16], anxiety, and depression [17, 18] are also associated with ON. Only a few studies investigated the relationship between personality and ON; however, neuroticism [19], perfectionism and narcissism [14], higher harm avoidance, higher transcendence, and lower self-directedness [20], persistence [21] and different personality disorder traits [22–25] are associated with higher orthorexic symptomatology.

Bratman (2017) [26] considers ON a phenomenon that could be divided into two stages: firstly, the person chooses to follow a healthy diet; later, healthy eating habits intensify and become problematic. Only this phase could be considered pathological: even if individuals adopt non-conventional diets, it is premature to consider this behavior as dysfunctional eating. However, not all diets are associated with ON; when related to other factors such as excessive concern for food, problems on social functioning, nutritional deficiencies, or weight loss, they could be considered disordered eating behavior [26]. Therefore, dieting could be considered essential in the etiology of ON, however this factor needs more investigation.

Motivations underlying the choice of a healthy diet have different roles on orthorexic tendencies: a recent study by Valente et al. (2020) [27] highlights that the most relevant motivation that predicts ON is the concern of developing chronic diseases and, less strongly, food intolerances or the pressures about beauty. Consequently, if adopting a healthy lifestyle and following a balanced diet is not considered a problematic behavior, distorted cognitions could lead to spend a great amount of time on rigid healthy habits, adhering to a strict diet. For this reason, ON can be considered a "paradox" of eating healthy [20]. Therefore, there could be orthorexic tendencies in people who follow a diet and also in those who are not following any diet.

To the best of our knowledge, no study has compared psychopathological dimensions of people who follow a diet with those who are not on a diet and that both highlight high orthorexic tendencies. The principal aim of this study is to understand differences in some psychopathological characteristics (EDs, obsessive–compulsive symptoms, perfectionism, anxiety, and depression) between groups on a diet with high/low orthorexic tendencies and people not on a diet with high orthorexic tendencies. People on a diet or not, both with higher orthorexic tendencies, are expected to show more psychopathological characteristics than those on a diet but with lower orthorexic tendencies. Moreover, another aim of this study is to compare these groups in maladaptive personality traits that could be related to ON; it is expected that orthorexic groups highlight more dysfunctional personality characteristics.

## Methods

### Participants

The sample of this study is part of a larger one whose results were previously published [11, 13]. The study was conducted among 133 adults belonging to three different groups: High Orthorexic Tendencies (HIGH) group, High Orthorexic Tendencies on Diet (HIGH-D) group, and Low Orthorexic Tendencies on Diet (LOW-D) group. Sociodemographic characteristics of three groups are reported in Table 1.

**Table 1:** Differences in sociodemographic characteristics between the HIGH, HIGH-D and LOW-D groups

	HIGH (1) *n* = 40	HIGH-D (2) *n* = 52	LOW-D (3) *n* = 41	F or CHI and sign (*p*)	Partial η^2^	Bonferroni post-hoc comparison (*p*)
**Age (years)**	20.78 (2.71)	42.68 (13.41)	47.87 (12.18)	71.48_(2)_ (< .001)	.53	**1 < 2;3 (< .001)**
School Years	14.77 (1.37)	14.16 (3.24)	14.21 (3.10)	.651_(2)_ (n.s.)	.01	n.s
**BMI (kg/ m**^**2**^**)** **(SD)**	21.30 (2.11)	24.10 (5.89)	25.37 (5.05)	7.69_(2)_ (< .001)	.11	**1 < 2;3 (< .05)**
Gender Female n (%)	20 (50%)	28 (53.85%)	24 (58.54%)	.597_(2)_ (n.s.)	-	-
Medical Diagnosis n (%)	2 (5%)	16 (30.8%)	9 (22%)	10.29_(2)_ (< .01)	-	-
Eating Habits n (%)						
Mostly Mediterranean Unconventional Diet Not Specificated Missing Values	37 (92.5%) 3 (7.5%)	2 (3.8%) 49 (94.2%) 1 (1.9%)	41 (100%)	109.53_(6)_ (< .001)	-	-
Eating Habits followed since n (%)						
Always followed From 11 to 15 years From 6 to 10 years From 1 to 5 years From 1 year or less Missing Values	27 (67.5%) 1 (2.5%) - 3 (7.5%) 2 (5.0%) 7 (17.5%)	2 (2.8%) 1 (1.9%) 6 (11.5%) 5 (9.6%) 35 (67.3%) 3 (5.8%)	- 2 (4.9%) 2 (4.9%) 3 (7.3%) 32 (78%) 2 (4.9%)	90.29_(10)_ (< .001)	-	-
Food Intolerance n (%)	7 (17.5%)	13 (25%)	3 (7.3%)	.84_(2)_ (n.s.)	-	-
Food Avoidance n (%)	23 (57.5%)	44 (84.6%)	24 (58.5%)	11.67_(2)_ (< .01)	-	-

*HIGH* High Orthorexic Tendencies, *HIGH-D* High Orthorexic Tendencies on Diet, *LOW-D* Low Orthorexic Tendencies on Diet, *BMI* Body Mass Index, *SD* Standard Deviation

The HIGH group was composed of 40 selected students enrolled using snowball sampling at the University of Padova (Italy). The original sample consisted of 302 students; the subjects of this group have been selected considering the 90^th^ percentile of the Eating Habits Questionnaire-21 (EHQ-21 [19]; the Italian version by [17]), corresponding to a cut-off of 50 points. Exclusion criteria were having a diagnosis of a psychiatric disorder, taking psychotropic drugs, and following a diet at the time of administration. In this sample, women and men are equally represented (males: *n* = 20; females: *n* = 20). The mean age of participants is 20.78 years (SD = 2.71), the mean of school years is 14.77 (SD = 1.37), and the mean of BMI is 21.30 kg/m^2^ (SD = 2.11). Participants did not report a medical diagnosis in 95% (*n* = 38) of cases. In addition, participants were required to point out which eating style was their usual way of eating. At the time of administration, 80% (*n* = 32) of the sample reported a mediterranean eating style, 7.5% (*n* = 3) a vegetarian eating style, 5.0% (*n* = 2) a vegan eating style, and 7.5% (*n* = 3) a not specific eating habit. Moreover, 67.5% (*n* = 27) of individuals reported that they have always followed these eating habits. 17.5% (*n* = 7) of the sample referred to food intolerances and 57.5% (*n* = 23) avoided some type of food.

The participants on a diet (*n* = 93) were recruited in a northern Italy dietician’s medical office. They were consecutively admitted and voluntarily followed a “zone diet” prescribed by the dietician. The “zone diet” is a food program that emphasizes low carbohydrate consumption, in order to reduce weight or improve mental and physical performance. The questionnaires were received by mail or by the doctor, were filled out at home and delivered to the dietician. No participant had a diagnosis of psychiatric disorder or was taking psychotropic drugs. Based on the EHQ-21 cut-off used for the HIGH group, two groups were identified: High Orthorexic Tendencies on Diet (HIGH-D) and Low Orthorexic Tendencies on Diet (LOW-D). Participants in the HIGH-D group obtained an EHQ-21 score higher than the 90^th^ percentile, while participants in the LOW-D group lower than the 90^th^ percentile.

The HIGH-D group (*n* = 52) scored over the EHQ-21 cut-off and it was considered a group on a diet with high orthorexic tendencies. Women are 53.85% (*n* = 28) of the participants. The mean age is 42.68 years (SD = 13.41), the mean of the school years is 14.16 (SD = 3.24), and the mean BMI is 24.10 kg/m^2^ (SD = 5.89). In this sample, 30.8% (*n* = 16) of participants reported a medical diagnosis. Regarding eating habits, 94.2% (*n* = 49) of the sample was following a zone diet at the time of administration and 3.8% (*n* = 2) a mediterranean one. Participants reported in 67.3% (*n* = 35) of cases that they have been following these eating habits for one year. Moreover, 25.0% (*n* = 13) of the sample reported food intolerance and 84.6% (*n* = 44) avoided some type of food.

The LOW-D group (*n* = 41) scored under the EHQ-21 cut-off and it was considered a group with low orthorexic tendencies. Women represent 58.54% (*n* = 24) of the sample. The mean age is 47.87 years (SD = 12.18), while the school years mean is 14.21 (SD = 3.10). BMI has a mean of 25.37 kg/m^2^ (SD = 5.05). Participants in this sample reported a medical diagnosis in 22% (*n* = 9) of cases. Regarding eating habits, all the people (*n* = 41) were following a zone diet at the time of administration. Participants reported that they have been following these eating habits for one year in 78.0% (*n* = 32) of cases. Moreover, 7.3% (*n* = 3) of the sample had a food intolerance and 58.8% (*n* = 24) avoided some type of food.

The purpose of this study was fully explained to participants before they voluntarily expressed their written and informed consent. Participants were invited to fill out a series of self-report questionnaires. Anonymity and confidentiality of the collected data were guaranteed. Some of these data were used in previous research [11, 13] with different aims.

This study was approved by the Department of General Psychology' Ethical Committee (University of Padova) and was conducted according to the Declaration of Helsinki.

### Measures

A demographic schedule was presented to all participants in order to collect data about gender, age, years of education, and BMI (calculated from self-reported information about weight and height). Moreover, the schedule asked participants to report if they were following any diet and some information on their eating habits. These information were in addition to those investigated with the administered questionnaires, thus they did not overlap with them.

Psychopathological characteristics were assessed with the following questionnaires.

The Eating Habits Questionnaire-21 (EHQ-21: [19]; the Italian version by [17]) has been used to investigate problems, thoughts, concerns, and emotions related to ON. The EHQ-21 is a self-report questionnaire composed of 21 items organized in three subscales: “Problems”, “Knowledge”, and “Feelings”. Item responses are expressed on a four-point Likert scale. Good internal consistency has been shown both in the original (0.82 < Cronbach’s α < 0.90) and in the Italian validation of the EHQ.

The current study highlighted good internal consistency for the EHQ total score and the three subscales (0.70 < Cronbach’s α < 0.81). The “Feelings” subscale was considered good (Cronbach’s α = 0.77) after “item 9” had been excluded.

The Personality Inventory for DSM-5 (PID-5; [28]; the Italian version by [29]) has been used to assess maladaptive personality traits proposed by the DSM-5. The PID-5 is a self-report questionnaire composed of 220 items organized in five Domains: “Negative Affectivity” (experience of negative emotions), “Detachment” (depression and mistrust), “Antagonism” (grandiosity and social withdrawal), “Disinhibition” (impulsivity and irresponsibility) and “Psychoticism” (eccentricity and perceptual problems). Item responses are expressed on a four-point Likert Scale. Good internal consistency has been shown both in the original (0.84 < Cronbach’s α < 0.96) and the Italian validation of the PID-5 for the 5 Domains.

The current study highlighted an excellent internal consistency for the five Domains of the PID-5 (0.90 < Cronbach’s α < 0.95).

The Eating Disorder Inventory-3 (EDI-3: [30]; the Italian version by [31]) has been used to assess symptoms and some psychological features related to EDs. The EDI-3 is a self-report questionnaire, and it is composed of 91 items organized in twelve subscales: "Drive for Thinness", "Bulimia", "Body Dissatisfaction", "Low Self-Esteem", "Personal Alienation", "Interpersonal Insecurity", "Interpersonal Alienation", "Interoceptive Deficits", "Emotional Dysregulation", "Perfectionism", "Asceticism", "Emotional Dysregulation", "Perfectionism", and "Maturity Fears". Item responses are expressed on a six-point Likert scale. Excellent internal consistency (0.90 < Cronbach’s α < 0.97) has been shown in the original validation of the EDI-3. Moreover, good internal consistency was highlighted in the Italian version (0.72 < Cronbach’s α < 0.94).

The current study highlighted a good internal consistency for the EDI-3 total score and its subscales (0.68 < Cronbach’s α < 0.95).

The Obsessive Compulsive Inventory-Revised (OCI-R: [32]; the Italian version by [33]) has been used to investigate symptoms of Obsessive–Compulsive Disorder. The OCI-R is a self-report questionnaire of 18 items organized in six subscales: "Washing", "Ordering", "Hoarding", "Mental Neutralizing", "Obsessing", and "Checking". Item responses are expressed on a five-point Likert scale. The original and the Italian version show good psychometric properties. The current study highlighted good internal consistency for the total scores and all subscales (0.73 < Cronbach’s α < 0.91).

The Multidimensional Perfectionism Scale (MPS: [34, 35]; the Italian version by [36]) has been used to investigate different perfectionistic traits. The MPS is a 35-item self-report questionnaire composed of six subscales: "Concern over Mistakes", "Personal Standards", "Parental Expectations", "Parental Criticism", "Doubting of Actions", and "Organization". Item responses are expressed on a five-point Likert scale. Good internal consistency has been shown both in the original (0.77 < Cronbach's α < 0.93) and the Italian version.

In the current study, all subscales showed good internal consistency (0.77 < Cronbach's α < 0.94).

The Beck Anxiety Inventory (BAI: [37]; the Italian version by [38]) has been used to assess the severity of anxiety symptoms. The BAI is a self-report questionnaire of 21 items expressed on a four-point Likert scale. The BAI displayed excellent internal consistency (Cronbach’s α = 0.92). The Italian version showed good internal consistency in a sample of students (Cronbach's α = 0.89).

Excellent internal consistency was highlighted in the current study (Cronbach's α = 0.93).

The Beck Depression Inventory-Second Edition (BDI-II: [39]; the Italian version by [40]) has been used to assess the severity of depression symptoms. The BDI-II is a self-report questionnaire of 21 items expressed on a four-point Likert scale. Excellent internal consistency was highlighted in a sample of university students (Cronbach's α = 0.93). In the Italian version, it has been highlighted good internal consistency both considering a sample of university students, patients with depression and a group of individuals extracted from the general population (0.80 < Cronbach's α < 0.87). The current study showed a good internal consistency (Cronbach's α = 0.89).

### Data analysis

All statistical analyses were performed using SPSS Statistic Version 25.0 software. Cronbach’s alpha was calculated for all scales and subscales of the self-report questionnaires.

A Chi-squared index was performed to investigate the differences between groups in gender.

In order to explore differences between groups, Multivariate ANOVA was conducted for other sociodemographic characteristics (age, school years, and BMI) and Multivariate analysis of covariance (MANCOVA). For the MANCOVAs, Fisher’s F and Partial Eta Squared were reported as effect sizes.

A Bonferroni corrected post-hoc comparison was performed to highlight differences between groups in sociodemographic characteristics and in the total score of questionnaires.

## Results

### Differences between groups in sociodemographic characteristics

Groups did not differ in gender (χ2 = 0.59; n.s.) and school years (F(2) = 0.65; η2 = 0.01; n.s.). However, groups differed in age (F(2) = 71.48; η2 = 0.53; *p* < 0.001): post-hoc Bonferroni highlighted that the HIGH group was younger than the other ones. The HIGH group had also the lowest BMI (F_(2)_ = 7.69; η2 = 0.11; *p* < 0.001). Groups differed in the reported number of medical diagnoses (χ2 = 10.29; *p* < 0.01); the HIGH group had the lowest one. There were also differences between groups in eating pattern (χ2 = 109.53; *p* < 0.001): HIGH group mostly followed a mediterranean eating habit, whereas HIGH-D and LOW-D groups had an unconventional diet. Participants of the HIGH group reported that they have always followed these eating habits, while participants of the HIGH-D and LOW-D groups have been following an unconventional diet for one year or less (χ2 = 90.29; *p* < 0.001). Moreover, the HIGH-D group avoided some food more frequently than others (χ2 = 11.67; *p* < 0.01). To control the impact of age and BMI in the differences between groups, MANCOVAs have been performed using those variables as covariates (Table 1).

### Differences between groups in EDs symptoms

Age was found to be a statistically significant covariate in terms of the group comparison of EDI total score (F_(1)_ = 14.85; *p* < . 001; η^2^ = 0.11), “Bulimia” (F_(1)_ = 6.49; *p* < 0.05; η^2^ = 0.05), “Body Dissatisfaction” (F_(1)_ = 9.97; *p* < 0.01; η^2^ = 0.07), “Low Self-Esteem” (F_(1)_ = 9.27; *p* < 0.01; η^2^ = 0.07), “Personal Alienation” (F_(1)_ = 10.60; *p* < 0.001; η^2^ = 0.08), “Interpersonal Alienation” (F_(1)_ = 4.58; *p* < 0.05; η^2^ = 0.04), “Interoceptive Deficits” (F_(1)_ = 9.93; *p* < 0.01; η^2^ = 0.07), “Emotional Dysregulation” (F_(1)_ = 4.41; *p* < 0.05; η^2^ = 0.03), and “Asceticism” (F_(1)_ = 5.57; *p* < 0.05; η^2^ = 0.04) subscales. BMI was found to be a statistically significant covariate in terms of the group comparison of “Bulimia” (F_(1)_ = 6.75; *p* < 0.05; η^2^ = 0.05) and “Body Dissatisfaction” (F_(1)_ = 7.30; *p* < 0.01; η^2^ = 0.06) subscales. In the EDI total score (F_(4)_ = 15.30; η^2^ = 0.33; *p* < 0.001), “Drive for Thinness” (F_(4)_ = 3.07; η^2^ = 0.09; *p* < 0.05), “Bulimia” (F_(4)_ = 8.45; η^2^ = 0.21; *p* < 0.001), “Body Dissatisfaction” (F_(4)_ = 4.39; η^2^ = 0.12; *p* < 0.01), “Low Self-Esteem” (F_(4)_ = 3.50; η^2^ = 0.10; *p* < 0.01), “Personal Alienation” (F_(4)_ = 6.34; η^2^ = 0.17; *p* < 0.001), “Interpersonal Insecurity” (F_(4)_ = 7.43; η^2^ = 0.19; *p* < 0.001), “Interpersonal Alienation” (F_(4)_ = 7.75; η^2^ = 0.20; *p* < 0.001), “Interoceptive Deficits” (F_(4)_ = 14.15; η^2^ = 0.31; *p* < 0.001), “Emotional Dysregulation” (F_(4)_ = 13.55; η^2^ = 0.30; *p* < 0.001), “Perfectionism” (F_(4)_ = 13.34; η^2^ = 0.30; *p* < 0.001), “Asceticism” (F_(4)_ = 22.73; η^2^ = 0.42; *p* < 0.001), and “Maturity Fears” (F_(4)_ = 6.06; η^2^ = 0.16; *p* < 0.001) subscales significant differences between groups were highlighted.

Considering the means of the three groups with the contribution of the covariates, the post-hoc Bonferroni tests highlighted that the HIGH group showed in “Emotional Dysregulation” (M = 6.00), “Perfectionism” (M = 7.13), and “Asceticism” (M = 7.12) subscales statistically significant higher scores both than the HIGH-D (respectively: M = 3.26 *p* < 0.05; M = 4.87 *p* < 0.05; M = 4.09; *p* < 0.001) and the LOW-D group (respectively: M = 2.13 *p* < 0.01; M = 4.14 *p* < 0.01; M = 2.69 *p* < 0.001). No differences between groups on a diet have been shown. Moreover, in the “Maturity Fears” subscale, the HIGH group (M = 10.05) had statistically significantly higher scores than the HIGH-D group (M = 6.43; *p* < 0.05). The LOW-D group did not show differences from any other groups (Table 2).

**Table 2:** Comparisons between the HIGH, HIGH-D and LOW-D groups

	**HIGH (1)** ***n* = 40**	**HIGH-D (2)** ***n* = 52**	**LOW-D (3)** ***n* = 41**	**F **_**(d.f.)**_	***p***	**Partial η**^**2**^	**Bonferroni post-hoc comparison (p)**	**α** ***N* = 133**
EHQ-21-TOT MEAN (SE)	53.49 (1.09)	57.81 (0.76)	44.51 (0.94)	42.98_(4)_	< .001	.58	**1 > 3 (*p* < .001)** **2 > 1;3(*p* < .01)**	.81
EHQ-21-PROBLEMS MEAN (SE)	25.73 (0.89)	26.83 (0.62)	19.14 (0.77)	26.07_(4)_	< .001	.46	**3 < 1;2 (*p* < .001)**	.77
EHQ-21-KNOWLEDGE MEAN (SE)	15.66 (0.45)	17.64 (0.32)	15.04 (0.39)	10.95_(4)_	< .001	.26	**2 > 1;3 (*p* < .01)**	.70
EHQ-21-FEELINGS MEAN (SE)	12.09 (0.41)	13.35 (0.28)	10.33 (0.35)	13.47_(4)_	< .001	.30	**3 < 1;2 (*p* < .01)**	.77
EDI-3-TOT MEAN (SE)	83.75 (7.07)	71.80 (4.94)	61.33 (6.12)	15.30_(4)_	< .001	.33	n.s	.95
EDI-3-DRIVE FOR THINNESS MEAN (SE)	7.47 (1.06)	7.13 (0.74)	5.28 (0.91)	3.07_(4)_	< .05	.09	n.s	.79
EDI-3-BULIMIA MEAN (SE)	5.41 (0.91)	3.61 (0.63)	2.73 (0.79)	8.45_(4)_	< .001	.21	n.s	.86
EDI-3-BODY DISSATISFACTION MEAN (SE)	8.24 (1.73)	12.91 (1.20)	11.33 (1.49)	4.39_(4)_	< .01	.12	n.s	.87
EDI-3-LOW SELF ESTEEM MEAN (SE)	3.11 (0.89)	4.89 (0.62)	4.65 (0.77)	3.50_(4)_	< .01	.10	n.s	.85
EDI-3-PERSONAL ALIENATION MEAN (SE)	4.87 (0.85)	4.87 (0.59)	4.89 (0.74)	6.34_(4)_	< .001	.17	n.s	.75
EDI-3-INTERPERSONAL INSECURITY MEAN (SE)	9.48 (1.07)	6.94 (0.74)	6.21 (0.92)	7.43_(4)_	< .001	.19	n.s	.82
EDI-3-INTERPERSONAL ALIENATION MEAN (SE)	7.57 (0.82)	7.12 (0.57)	5.81 (0.71)	7.75_4)_	< .001	.20	n.s	.71
EDI-3-INTEROCEPTIVE DEFICITS MEAN (SE)	7.33 (1.02)	5.70 (0.71)	3.92 (0.88)	14.15_(4)_	< .001	.31	n.s	.85
EDI-3-EMOTIONAL DYSREGULATION MEAN (SE)	6.00 (0.77)	3.26 (0.54)	2.13 (0.66)	13.55_(4)_	< .001	.30	**1 > 2;3 (*p* < .05)**	.82
EDI-3-PERFECTIONISM MEAN (SE)	7.13 (0.66)	4.87 (0.46)	4.14 (0.57)	13.34_(4)_	< .001	.30	**1 > 2;3 (*p* < .05)**	.71
EDI-3-ASCETICISM MEAN (SE)	7.12 (0.62)	4.09 (0.44)	2.69 (0.54)	22.73_(4)_	< .001	.42	**1 > 2;3 (*p* < .01)**	.68
EDI-3-MATURITY FEARS MEAN (SE)	10.05 (1.02)	6.43 (0.71)	7.56 (0.88)	6.06_(4)_	< .001	.16	**1 > 2 (*p* < .05)**	.73
OCI-R-TOT MEAN (SE)	16.75 (2.58)	17.49 (1.80)	11.04 (2.24)	3.96_(4)_	< .01	.11	n.s	.91
MPS-TOT MEAN (SE)	97.41 (4.58)	106.33 (3.19)	89.99 (3.96)	6.38_(4)_	< .001	.17	**2 > 3 (*p* < .01)**	.94
BAI-TOT MEAN (SE)	12.32 (1.99)	11.40 (1.39)	4.32 (1.73)	6.77_(4)_	< .001	.18	**3 < 1;2 (*p* < .05)**	.93
BDI-II-TOT MEAN (SE)	8.15 (1.56)	9.90 (1.09)	5.43 (1.35)	4.97_(4)_	< .001	.14	**2 > 3 (*p* < .05)**	.89
PID-5-NEGATIVE-AFFECTIVITY MEAN (SE)	23.79 (2.58)	25.27 (1.80)	18.29 (2.23)	7.49_(4)_	< .001	.20	**2 > 3 (*p* < .05)**	.94
PID-5-DETACHMENT MEAN (SE)	20.44 (2.33)	20.25 (1.63)	13.02 (2.02)	4.64_(4)_	< .01	.13	**2 > 3 (*p* < .01)**	.92
PID-5-ANTAGONISM MEAN (SE)	19.48 (1.94)	11.51 (1.35)	6.85 (1.68)	9.93_(4)_	< .001	.24	**1 > 2;3 (*p* < .01)**	.92
PID-5-DISINHIBITION MEAN (SE)	19.76 (1.85)	15.61 (1.29)	11.91 (1.60)	9.63_(4)_	< .001	.24	**1 > 3 (*p* < .05)**	.90
PID-5-PSYCHOTICISM MEAN (SE)	28.08 (2.96)	21.34 (2.07)	12.51 (2.56)	11.34_(4)_	< .001	.27	**3 < 1;2 (*p* < .05)**	.95

*HIGH* High Orthorexic Tendencies, *HIGH-D* High Orthorexic Tendencies on Diet, *LOW-D* Low Orthorexic Tendencies on Diet, *EHQ-21* Eating Habits Questionnaire-21, *EDI-3* Eating Disorder Inventory-3, *PID-5* Personality Inventory for DSM-5, *OCI-R* Obsessive Compulsive Inventory-*Revised, MPS* Multidimensional Perfectionism Scale, *BAI* Beck Anxiety Inventory, *BDI-II* Beck Depression Inventory-II, *SE* Standard Error, *n.s.* Not statistically significant, *d.f.* Degrees of freedom, *α* Cronbach’s Alpha coefficient

### Differences between groups in obsessive–compulsive symptoms and perfectionistic traits

No statistical significance of the covariates (age and BMI) was highlighted on the OCI-R and MPS questionnaires. Post-hoc Bonferroni did not highlight differences in Obsessive–Compulsive symptoms between HIGH and on diet groups.

Differences between groups were highlighted in MPS total score (F_(4)_ = 6.38; η^2^ = 0.17; *p* < 0.001).

The post-hoc Bonferroni tests highlighted that, in the MPS total score, the HIGH-D group (M = 106.33) scored statistically significantly higher than the LOW-D group (M = 89.99; *p* < 0.01). No differences between the HIGH and on diet groups have been shown in perfectionistic traits (Table 2).

### Differences between groups in anxiety and depression

No statistical significance of age and BMI was highlighted on BAI and BDI total score. In the.

BAI total score, significant differences between groups were highlighted (F_(4)_ = 6.77; η^2^ = 0.18; *p* < 0.001). The post-hoc Bonferroni tests highlighted that, on the BAI total score, the LOW-D group (M = 4.32) showed statistically significant lower scores both than the HIGH (M = 12.32; *p* < 0.05) and the HIGH-D (M = 11.40; *p* < 0.01) group. No differences between the HIGH and HIGH-D have been highlighted in anxiety features.

In the BDI total score significant differences between groups were highlighted (F_(4)_ = 4.97; η^2^ = 0.14; *p* < 0.001). The post-hoc Bonferroni tests highlighted on the BDI total score that the HIGH-D group (M = 9.90) showed statistically significantly higher scores than the LOW-D (M = 5.43; *p* < 0.05) group. The HIGH group did not show differences from any other groups (Table 2).

### Differences between groups in maladaptive personality traits

Age was found to be a statistically significant covariate in terms of the group comparison of “Negative Affectivity” scores (F_(1)_ = 5.85; *p* < 0.05; η^2^ = 0.05) PID-5 Domains. The covariate BMI is statistically significant compared to the dependent variable of the “Psychoticism” (F_(1)_ = 4.70; *p* < 0.05; η^2^ = 0.04) PID-5 Domains.

In the PID-5 Domains of “Negative Affectivity” (F_(4)_ = 7.49; η^2^ = 0.20; *p* < 0.001), “Detachment” (F_(4)_ = 4.64; η^2^ = 0.13; *p* < 0.01), “Antagonism” (F_(4)_ = 9.93, η^2^ = 0.24, *p* < 0.001), “Disinhibition” (F_(4)_ = 9.63; η^2^ = 0.24; *p* < 0.001), and “Psychoticism” (F_(4)_ = 11.34; η^2^ = 0.27; *p* < 0.001) significant differences between groups were highlighted.

When the means of the three groups were considered with the contribution of the covariates, the post-hoc Bonferroni tests highlighted that, in the “Negative Affectivity” Domain, the HIGH-D group (M = 25.27) had a statistically significant higher score than the LOW-D group (M = 18.29; *p* < 0.05). The same pattern has been shown in the “Detachment” Domain, where HIGH-D (M = 20.25) scored higher than the LOW-D group (M = 13.02; *p* < 0.01). For these Domains, no differences between the HIGH group and groups on a diet have been highlighted. In “Antagonism” Domain HIGH group (M = 19.48) showed statistically significant higher scores both than the HIGH-D (M = 11.51; *p* < 0.01) and LOW-D (M = 6.85; *p* < 0.001) groups. No differences between groups on a diet have been shown. Regarding “Disinhibition” Domain, the HIGH group (M = 19.76) scored statistically significantly higher than the LOW-D group (M = 11.91; *p* < 0.05). The HIGH-D group did not show differences from any other groups. In “Psychoticism” Domain, the LOW-D group (M = 12.51) scored statistically significantly lower both than the HIGH group (M = 28.08; *p* < 0.01) and the HIGH-D group (M = 21.34; *p* < 0.01) (Table 2).

## Discussion

The principal aim of this study was to investigate if people on a diet with high orthorexic tendencies (HIGH-D) showed differences in psychopathological characteristics in comparison with people not on a diet with high orthorexic tendencies (HIGH) and with people on a diet with low orthorexic tendencies (LOW-D). For this purpose, ON was assessed with the EHQ and the sample was divided into three groups: the High Orthorexic Tendencies (HIGH) group, High Orthorexic Tendencies on Diet (HIGH-D) group, and the Low Orthorexic Tendencies on Diet (LOW-D) group.

Considering the EHQ “Knowledge” subscale, the HIGH-D group scored significantly higher both than the HIGH and the LOW-D groups. Regarding the “Problems” and “Emotions” subscales, there were no differences between groups with high orthorexic tendencies (HIGH and HIGH-D), and both scored higher than the LOW-D group.

These results are consistent with the literature that suggests diet as a possible risk factor for Orthorexia Nervosa [11, 41]. Nevertheless, groups of people with high orthorexic tendencies -both on diet and not on diet- show differences in orthorexic features, personality traits, eating-disorders characteristics, and in other aspects; as a consequence, diet alone can not explain the tendencies described above [26]. For these reasons, we investigated some psychopathological aspects related to ON, which could have a relationship with the construct.

Regarding perfectionism, the HIGH-D group showed higher scores than the LOW-D group. ON symptomatology is related to non-adaptive perfectionism [14, 15], and it concerns high personal standards and rigid organization that could explain the strict adherence to healthy eating. Moreover, people on a diet could be more likely to expose themselves to rigorous dietary rules and perfectionism could impact on orthorexic tendencies in this group [11]. Furthermore, the HIGH-D group showed higher levels of depression than the LOW-D group; the excessive focus on healthy food could compromise social functioning and lead to a deflection of mood [17].

The HIGH group showed higher scores in EDI “Emotional Dysregulation”, “Perfectionism”, and “Asceticism” subscales than the other two groups and higher scores in the “Maturity Fears” subscale than the LOW-D group. Those dimensions correlate with the symptomatology of EDs [42], but they do not represent nuclear aspects of it; our results highlight a possible coexistence between high orthorexic tendencies and some secondary, but still problematic, aspects of EDs.

Both the HIGH-D group and the HIGH group had greater anxious symptomatology than the LOW-D group; this result is consistent with studies that highlight the presence of higher anxiety in orthorexic groups [18]. Those groups did not show differences in Obsessive–compulsive features, as reported in recent literature that distinguish these from ON [13].

The second aim of this study was to understand maladaptive personality traits in orthorexic groups that were not much investigated in the literature. As expected, orthorexic groups had more dysfunctional personality traits than the LOW-D group; moreover, we highlighted different personality traits between HIGH-D and HIGH groups [22, 25].

Particularly, the HIGH-D group had higher scores in “Negative Affectivity” and “Detachment” domains than the LOW-D group. “Negative affectivity” is a trait characterized by intense and frequent experiences of high levels of negative emotions (e.g., anxiety, depression, guilt/shame, worry, anger) and associated behavioral and interpersonal manifestations [5, 28]. This maladaptive dimension of personality is higher in orthorexic people on a diet and it could explain intense negative emotions felt whenever it fails the strict adherence to a healthy diet [7]. “Detachment” refers to avoiding socioemotional experiences, lack of interpersonal relationships with others, difficulties in giving/receiving empathy, and limited hedonic capacity [5, 28]. Traits related to this maladaptive personality dimension occur in conjunction with beliefs and problems related to higher orthorexic tendencies in the HIGH-D group; the attitude of superiority and teaching of eating habits to others could lead to withdrawal, isolation and social impairment [2].

Also, the HIGH group could suffer from social compromission, as highlighted in high levels of “Antagonism”. This dimension involves an exaggerated sense of self-importance, grandiosity, attention-seeking and lack of empathy toward others, which includes both the unawareness of the needs of others and the tendency to use others for one's advantage [5, 28]. Moreover, the HIGH group had higher levels of “Disinhibition” than the group with lower orthorexic tendencies; this is the first time that the maladaptive personality dimension of “Disinhibition'' and the ON show an association. This relationship is plausible; however, it deserves further investigation.

Moreover, both orthorexic groups highlighted higher levels of “Psychoticism” than the LOW-D group. “Psychoticism” is a maladaptive personality trait characterized by incongruent, bizarre, eccentric, or unusual thoughts and by cognitive and perceptual dysregulation (e.g., depersonalization, derealization, and dissociative experiences) [5, 28]. As also shown in EDs, people with high orthorexic tendencies could present bizarre and unusual beliefs about nutrition and healthy food and cognitive distortions/maladaptive thoughts that could explain the excessive fixation on eating healthy [43]. 

A more restrictive diet and a diet that involves many changes in eating habits could represent a risk factor for ON [11, 41]. In our study, the HIGH-D reported more food avoidance than the other groups; nevertheless, the HIGH and the LOW-D did not show differences. Results highlighted food avoidance in 57.5% of participants of the HIGH group and 58.5% of the LOW-D group. Even if the LOW-D group was following a diet that excluded some foods, the HIGH group was not following any diet that could explain this avoidance behavior. Patients with EDs restrict the diet variety [44], and this behavior is in relationship with the worst outcome in the post-EDs-treatment period [45]. Thus, the HIGH group could be considered at risk for developing disordered eating habits, confirming the previous findings that considered ON as part of the EDs spectrum. However, in the onset of ON symptomatology, it is also important to highlight the role of other psychological features. In line with the hypothesis of the present study, groups with higher orthorexic tendencies showed more psychopathological characteristics and more maladaptive personality traits than the group with lower orthorexic tendencies.

The most important limit of this study is that it is cross-sectional; therefore, it does not provide any information about the evolution of Orthorexia Nervosa through time. Longitudinal studies on each group could be important to understand if ON could be considered a prodromal condition of a pathological eating habit and if it could be an outcome of dieting in subjects most at risk of developing it. Future studies should investigate groups from the general population and other groups on a diet potentially at risk of ON, to pay preventive attention to them.

Another limit is represented by the lack of information on the reasons underlying the choice of a diet or eating habits: this aspect could be related to ON and deserves more investigation.

The present study did not investigate the socio-economic status of the participants, thus its effect on the main variables could not be tested. Moreover, even if analysis controlled differences in age, future studies should make more specific comparisons in similar groups.

Finally, this study is based on self-reported questionnaires: it could be important to evaluate psychopathological characteristics associated with ON with clinical evaluation by health care professionals.

## Conclusions

This study put in evidence that people with high orthorexic tendencies could present dysfunctional eating habits even if they are not following any diet. Diet alone can not explain ON, but only considering other psychopathological factors. Nevertheless, diet could also be followed in a healthy and non-dysfunctional way.

## Data Availability

The datasets generated and/or analyzed during the current study are not publicly available due their containing information that could compromise the privacy of research participants but are available from the corresponding author on reasonable request.
